# PAK4 confers cisplatin resistance in gastric cancer cells via PI3K/Akt- and MEK/ERK-dependent pathways

**DOI:** 10.1042/BSR20130102

**Published:** 2014-03-04

**Authors:** Xueqiong Fu, Jiarui Feng, Duan Zeng, Yu Ding, Changshou Yu, Bing Yang

**Affiliations:** *Department of Gastroenterology, Longgang District Central Hospital of Shenzhen, Shenzhen 518116, People’ Republic of China; †Department of Gastroenterology, The Fifth Hospital of Wuhan, Wuhan 430050, People’ Republic of China

**Keywords:** cisplatin (CDDP), p21-activated kinase 4 (PAK4), MEK/ERK, PI3K/Akt, gastric cancer, Akt, protein kinase B or PKB, CDDP, cisplatin, ERK, extracellular-signal-regulated kinase, HRP, horseradish peroxidase, MEK, MAPK (mitogen-activated protein kinase)/ERK (extracellular-signal-regulated kinase) kinase, PAK, p21-activated kinase, PAK4, p21-activated kinase 4, PI3K, phosphoinositide 3-kinase, siRNA, small interfering RNA

## Abstract

CDDP [cisplatin or cis-diamminedichloroplatinum(II)] and CDDP-based combination chemotherapy have been confirmed effective against gastric cancer. However, CDDP efficiency is limited because of development of drug resistance. In this study, we found that PAK4 (p21-activated kinase 4) expression and activity were elevated in gastric cancer cells with acquired CDDP resistance (AGS/CDDP and MKN-45/CDDP) compared with their parental cells. Inhibition of PAK4 or knockdown of PAK4 expression by specific siRNA (small interfering RNA)-sensitized CDDP-resistant cells to CDDP and overcome CDDP resistance. Combination treatment of LY294002 [the inhibitor of PI3K (phosphoinositide 3-kinase)/Akt (protein kinase B or PKB) pathway] or PD98509 {the inhibitor of MEK [MAPK (mitogen-activated protein kinase)/ERK (extracellular-signal-regulated kinase) kinase] pathway} with PF-3758309 (the PAK4 inhibitor) resulted in increased CDDP efficacy compared with LY294002 or PD98509 alone. However, after the concomitant treatment of LY294002 and PD98509, PF-3758309 administration exerted no additional enhancement of CDDP cytotoxicity in CDDP-resistant cells. Inhibition of PAK4 by PF-3758309 could significantly suppress MEK/ERK and PI3K/Akt signalling in CDDP-resistant cells. Furthermore, inhibition of PI3K/Akt pathway while not MEK/ERK pathway could inhibit PAK4 activity in these cells. The *in vivo* results were similar with those of *in vitro*. In conclusion, these results indicate that PAK4 confers CDDP resistance via the activation of MEK/ERK and PI3K/Akt pathways. PAK4 and PI3K/Akt pathways can reciprocally activate each other. Therefore, PAK4 may be a potential target for overcoming CDDP resistance in gastric cancer.

## INTRODUCTION

Gastric cancer is one of the most common causes of cancer-related mortality worldwide [1]. CDDP [cisplatin or cis-diamminedichloroplatinum(II)] is the most frequently used chemotherapeutic agent for various types of advanced cancer. In a phase II study, the response rate of CDDP treatment against advanced gastric cancer was 22% [2] and cases of complete remission were rare. Some CDDP-based combination chemotherapy have been used to improve the treatment outcomes and shown high response rates [3,4]. However, intrinsic or acquired resistance to CDDP reduces its efficacy and is a major obstacle for the effective treatment of cancers.

The small GTPases, i.e. Ras, Rho, Rac and Cdc42 contribute to many hallmarks of cancer. The PAKs (p21-activated kinases) are among the best characterized downstream effectors of Rac and Cdc42. PAKs are a family of serine/threonine protein kinases consist of six isoforms (PAK1-6) [5]. Increasing evidences have confirmed that PAKs are closely correlated with cancer development. PAKs are overexpressed and/or hyperactivated in several human tumours such as breast cancer, colon cancer, lung cancer and gastric cancer [6,7]. For cell proliferation, PAKs are essential for Ras-induced cell cycle progression [8]. PAKs have also been implicated in other cellular processes relevant to tumourigenesis, including angiogenesis [9], epithelial–mesenchymal transition [10] and metabolism [11,12]. Especially for PAK4, it is strongly implicated in oncogenic transformation and its activity is required for Ras-driven anchorage-independent growth in various cancer cell lines [13]. Besides, there is a novel association between Gab1 and PAK4, and PAK4 is a key intergrator of cancer cell migration and invasive growth downstream from the Met receptor [14]. PAK4 was found to be overexpressed in metastatic gastric cancer patients, implicating a role of PAK4 in gastric cancer metastasis [15]. Li et al. [7] reported that DGCR6L, a novel PAK4-interacting protein, regulated PAK4-mediated migration of human gastric cancer cells via LIMK1. Potent inhibition of PAK4 by LCH-7749944 suppressed invasion of human gastric cancer cells [16]. Metastasis and chemoresistance in cancer are linked phenomena. However, whether PAK4 plays a role in chemoresistance in cancer cells remains undefined.

This study was designed to explore the potential relationship between PAK4 and CDDP resistance in gastric cancer cells. Our results demonstrate that PAK4 confers CDDP resistance in gastric cancer cells via the activation of MEK/ERK (MAPK or mitogen-activated protein kinase/extracellular-signal-regulated kinase) and PI3K/Akt (phosphoinositide 3-kinase/protein kinase B or PKB) pathways. Therefore, PAK4 may be a potential target for overcoming CDDP resistance in gastric cancer therapy.

## MATERIALS AND METHODS

### Materials

CDDP was from Sigma; LY294002 (PI3K inhibitor) was obtained from Merk. Cell culture reagents were obtained from Invitrogen. The PAK4 antibody and HRP (horseradish peroxidase)-labelled anti-rabbit secondary antibody were purchased from Cell Signaling Technology. All other reagents were from Sigma unless stated otherwise.

### Cell lines and cell culture

The gastric cancer cell lines (AGS and MKN-45) were from the American Tissue Culture Collection (ATCC, USA) and cultivated according to the recommendation of the supplier, in the RPMI-1640 medium containing 10% (v/v) FBS, penicillin (100 units/ml) and streptomycin (100 μg/ml) at 37°C and 5% (v/v) CO_2_.

### Establishment of CDDP-resistant sublines from the AGS and MKN-45 cell lines

The CDDP-resistant cell sublines were developed by continuous exposure to CDDP starting at 0.1 μg/ml and increasing in a stepwise manner to 1 μg/ml according to a previous report [17]. The resistant sublines were maintained in the medium containing CDDP for more than 10 months. The CDDP-resistant sublines were named AGS/CDDP and MKN-45/CDDP throughout the text. Experiments with these sublines were performed after maintenance in the CDDP-free medium for 2–3 weeks.

### MTT assay

Approximately 5×10^4^ cells in 100 μl of serum-free RPMI-1640 medium were cultured in 96-well plates and incubated overnight. Then cells were treated with various agents for 48 h. After then, 20 μl of MTT labelling reagent (5 mg/ml) was added to the designated wells, and cells were incubated at 37°C for 4 h. Then, the supernatant was removed, and 150 μl DMSO was added to the designated wells. After the plates were incubated at 37°C for 15 min, the absorbency was measured with a micro ELISA reader (Bio-Tek) at a wavelength of 570 nm.

### Western blotting

Cell lysates were separated by SDS/PAGE in 10% (w/v) Tris–glycine gels and transferred to a nitrocellulose membrane. For analysis of PAK4 and phosphor-PAK4, blots were probed with their specific antibodies (diluted with 5% (w/v) BSA to 1: 1000). For analysis of Akt, ERK and their phosphorylated forms, blots were probed with their specific antibodies (diluted with 5% BSA to 1: 500). Non-phosphorylated total Akt and ERK bands were chosen as loading control for Akt and ERK activation, respectively. Then, membranes were probed with HRP-labelled anti-rabbit secondary antibody (diluted with 5% BSA to 1: 1000). Antibody binding was detected by enhanced enhanced ECL detection kit (UK Amersham International plc).

### siRNA (small interfering RNA) transfection

Gene silencing by RNA interference (siRNA) was used to down-regulate PAK4 expression in AGS/CDDP and MKN-45/CDDP cells. PAK4 siRNA (sc-39060, Cell Santa Cruz Biotechnology) that specifically inhibits the expression of PAK4 was used. siRNA (50 nM) was transfected into cells using Lipofectamine RNAiMAX (Invitrogen). The knockdown of target genes was confirmed by Western blotting after 48 h. Controls were transfected with non-specific siRNA and grown under similar conditions.

### Xenograft studies

All the procedures involving animals were according to the NIH Guide for the Care and Use of Laboratory Animals and local institutional ethical guidelines for animal experiment. Mice were anaesthetized with 0.2 ml of saline containing 25 mg/ml ketamine hydrochloride and 2.5 mg/ml xylazine. AGS and AGS/CDDP cells (~1×10^7^ cells) were subcutaneously inoculated into the right flank of 6-week-old nude mice. Treatments were started when tumours reached 80–100 mm^3^. All the mice were received CDDP administration (10 mg/kg per two times per week, i.p.). Animals were randomized to receive above treatments. Tumour sizes were calculated with the formula: (mm^3^)=(L×W^2^)×0.5.

### Immunohistochemistry

When tumours reached 80–100 mm^3^, tissues harvested from AGS, AGS/CDDP and AGS/CDDP/PAK4 siRNA tumours were fixed in 10% (v/v) formaldehyde overnight and embedded in paraffin. After deparaffinization, hydration and blockage of endogenous peroxidase, sections were pretreated by microwave for 20 min in 10 mM sodium citrate buffer for antigen retrieval. The slides were blocked with 5% (v/v) goat serum at room temperature for 1 h and then incubated with antibody against p-AKT, p-ERK or PAK4 (Cell Signaling Technology) (all diluted to 1:100) overnight at 4°C, followed by incubation with HRP-conjugated secondary antibodies for 1 h at room temperature. Slides were counterstained with haematoxylin, then photographed and converted to a digital image using light microscopy equipped with camera. For determining Ki67 staining, when tumours reached 80–100 mm^3^, mice were received 2-week CDDP treatment, then tumour tissues were harvested and incubated overnight with anti-Ki67 Ab (1:100) (cat. no. M7240, Dako dilution at 1:100). Next day, the reaction was detected using chromogen according to the manufacturer's instruction (Dako).

### Statistical analysis

Data were statistically analysed using Sigma Plot software (Jandel Scientific). Data were analysed by Unpaired Student's *t* test at a significance level *P* value of <0.05 and were presented as means±S.D.

## RESULTS

### PAK4 expression and activity are elevated in CDDP-resistant gastric cancer cells

**Figure 1:**
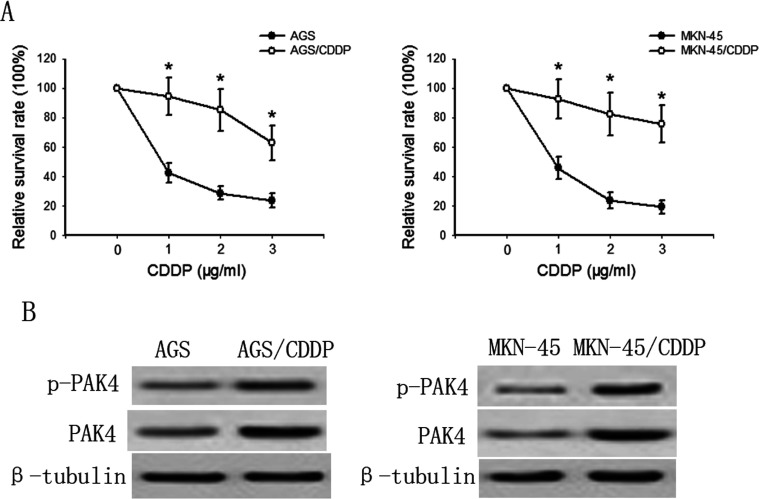
CDDP resistance correlates with elevated PAK4 (**A**) The CDDP (1 μg/ml) cytotoxicity in AGS/CDDP and MKN-45/CDDP cells and their parental cells was measured by MTT assay after 48-h incubation. Bars are mean±S.D. from four independent experiments. *P<0.05 versus DMSO control. (**B)** The differential expression pattern of phosphorylated and total PAK4 in CDDP-resistant gastric cancer cells (AGS/CDDP and MKN-45/CDDP) and their parental cells were determined by Western blotting.

To explore the effect of PAK4 on CDDP resistance in gastric cancer cells, we first established two CDDP-resistant gastric cancer cell lines-AGS/CDDP and MKN-45/CDDP by continuous exposure to CDDP starting at 0.1 μg/ml and increasing in a stepwise manner to 1 μg/ml. As shown in Figure 1(A), The results showed that parental AGS and MKN-45 cells were sensitive to CDDP (1–5 μg/ml), whereas established A549/CDDP cells were relatively resistant to CDDP treatment. Figure 1(B) showed that both the total and phosphorylated PAK4 levels in AGS/CDDP and MKN-45/CDDP cells were significantly higher than those in their parental cells, respectively. These results suggest a potential role of PAK4 in CDDP resistance development.

### Inhibition of PAK4 activity sensitizes AGS/CDDP and MKN-45/CDDP cells to CDDP

**Figure 2:**
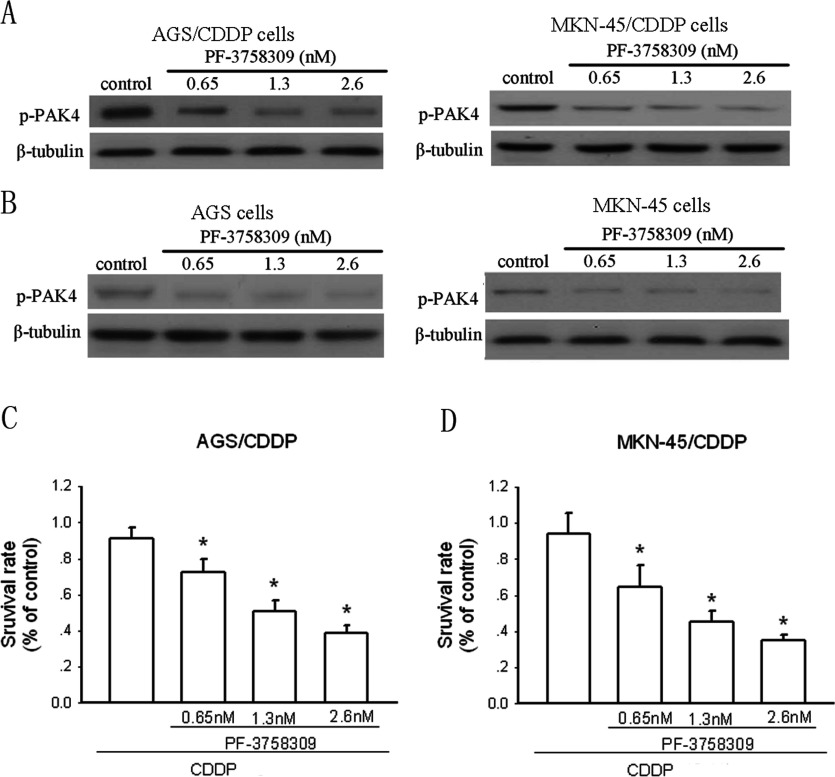
Inhibition of PAK4 activity increases CDDP efficacy in CDDP-resistant gastric cancer cells (**A**) The effect of the PAK4 inhibitor PF-3758309 at the indicated concentrations on p-PAK4 expression in AGS/CDDP and MKN-45/CDDP cells determined by Western blotting after 48-h incubation. (**B**) The effect of PF-3758309 at the indicated concentrations on p-PAK4 expression in AGS and MKN-45 cells determined by Western blotting. The effect of the PAK4 inhibitor PF-3758309 at the indicated concentrations on growth of AGS/CDDP (**C**) and MKN-45/CDDP (**D**) cells was assayed by MTT after 48-h incubation. The effect of the PAK4 inhibitor PF-3758309 at the indicated concentrations on growth of AGS/CDDP (**C**) and MKN-45/CDDP (**D**) cells was assayed by MTT after 48-h incubation. Bars are mean±S.D. from four independent experiments,*P<0.05.

Next, we used the PAK4 inhibitor PF-3758309 to determine the role of PAK4 in CDDP resistance in gastric cancer cells. PF-3758309 potently suppresses PAK4 activity confirmed by Murray et al. [17]. We performed Western blotting assay to confirm the effect of PF-3758309 on p-PAK4 levels first. The results showed that after 48-h treatment, PF-3758309 could significantly reduce the p-PAK4 levels in AGS/CDDP and MKN-45/CDDP cells in a dose-dependent manner (Figure 2A). Such effects also existed in AGS and MKN-45 cells (Figure 2B). Figure 2(C) shows that AGS/CDDP cells were insensitive to CDDP (1 μg/ml). PF-3758309 administration could dose-dependently increase CDDP efficacy in AGS/CDDP cells after 48-h incubation. Similar results were also obtained in MKN-45/CDDP cells (Figure 2D).

### Knockdown of PAK4 expression reverses CDDP resistance in gastric cancer cells

**Figure 3:**
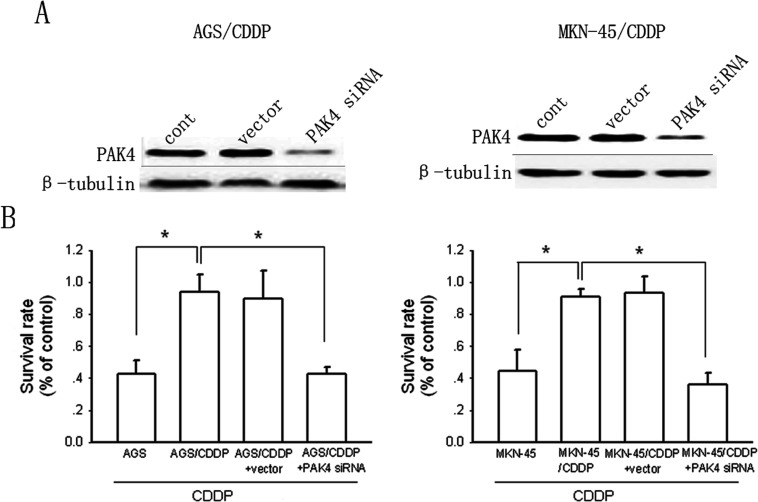
Knockdown of PAK4 reverses CDDP resistance (**A**) The effects of specific siRNA and non-silencing siRNA on PAK4 expression were determined by Western blotting in AGS/CDDP and MKN-45/CDDP cells. (**B**) The influence of PAK4 knockdown on cell growth after 48-h incubation was detected by MTT. Bars are mean±S.D. from five independent experiments,*P<0.05.

To confirm the role of PAK4 in CDDP resistance, we used the specific PAK4 siRNA to inhibit PAK4 expression. As shown in Figure 3(A), the PAK4 siRNA (50 nM) substantially reduced PAK4 expression in AGS/CDDP cells and MKN-45/CDDP cells by ~89 and 85% compared with control after 48-h treatment, respectively. The non-silencing siRNA (50 nM) had no effect on PAK4 expression. As shown in Figure 3(B), knockdown of PAK4 expression reversed CDDP resistance as the cytotoxicity of CDDP in PAK4 siRNA-tranfected AGS/CDDP cells and MKN-45/CDDP cells was comparable with that in AGS and MKN-45 cells after 48-h incubation. The non-silencing siRNA exerted no effects on CDDP cytotoxicity in AGS/CDDP cells and MKN-45/CDDP cells.

### PAK4 confers CDDP resistance in gastric cancer cells via MEK/ERK- and PI3K/Akt-dependent pathways

**Figure 4:**
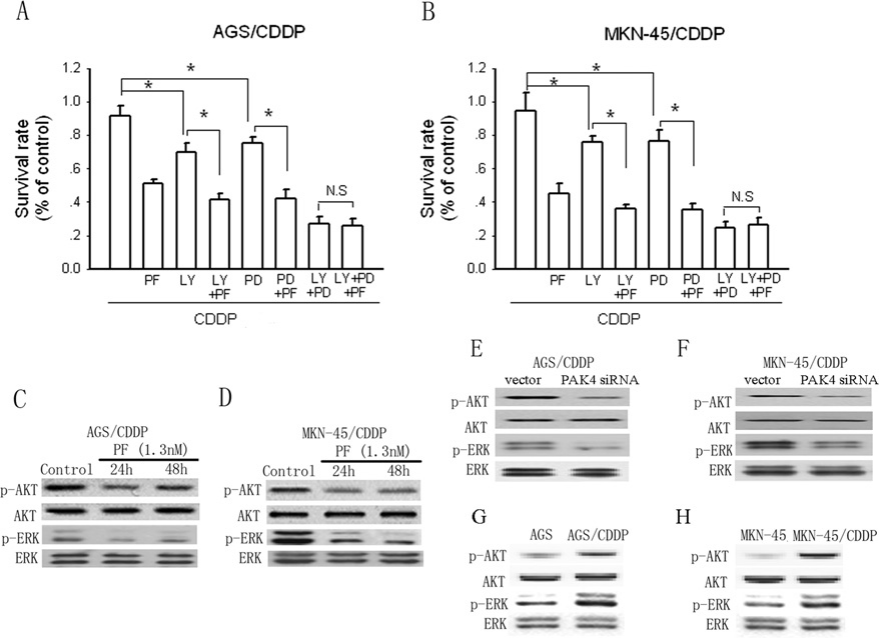
The involvement of MEK/ERK- and PI3K/Akt-dependent pathways in PAK4-induced CDDP resistance in gastric cancer cells (**A** and **B**) The effects of the PI3K/Akt pathway inhibitor LY294002 (LY) or the MEK/ERK inhibitor PD98509 (PD) alone or combination with the PAK4 inhibitor PF-3758309 (PF) on CDDP cytotoxicity in AGS/CDDP and MKN-45/CDDP cells detected by MTT assay after 48-h incubation, respectively. Bars are mean±S.D. from four to six independent experiments,*P<0.05. (**C** and **D**) The effect of the PAK4 inhibitor PF-3758309 (PF) on the activity of MEK/ERK and PI3K/Akt pathways represented by the level of phosphorylated ERK and Akt determined by Western blotting at the indicated time points in AGS/CDDP and MKN-45/CDDP cells, respectively. (**E** and **F**) The effect of the PAK4 siRNA on the activity ERK and Akt determined by Western blotting in AGS/CDDP and MKN-45/CDDP cells, respectively. (**G** and **H**) The differential pattern of Akt and ERK activities in CDDP-resistant gastric cancer cells (AGS/CDDP and MKN-45/CDDP) and their parental cells were determined by Western blotting.

To explore the mechanism underlying PAK4-induced CDDP resistance in gastric cancer cells, the PI3K/Akt pathway inhibitor LY294002 and the MEK/ERK inhibitor PD98509 were used. As shown in Figure 4(A), LY294002 (30 μM) or PD98509 (30 μM) treatment alone enhanced CDDP cytotoxicity in AGS/CDDP cells. Combination treatment of LY294002 or PD98509 with PF-3758309 (1.3 nM) resulted in increased CDDP efficacy compared with LY294002 or PD98509 alone. However, when both the MEK/ERK and PI3K/Akt pathways were inhibited by concomitant treatment of LY294002 and PD98509, PF-3758309 administration exerted no additional enhancement of CDDP cytotoxicity in AGS/CDDP cells. Similar results were also obtained in MKN-45/CDDP cells (Figure 4B). Figures 4(C) and 4(D) show that PF-3758309 (1.3 nM) treatment could significantly suppress MEK/ERK and PI3K/Akt signalling at the indicated time points in AGS/CDDP and MKN-45/CDDP cells, respectively. Knockdown PAK4 with siRNA also led to substantial suppression of AKT and ERK activities in AGS/CDDP (Figure 4E) and MKN-45/CDDP cells (Figure 4F). Furthermore, the activities of Akt and ERK in AGS/CDDP were much high than those in AGS cells (Figure 4G). Similar results were also obtained in MKN-45/CDDP cells (Figure 4H). Thus, these results demonstrate that PAK4 induces CDDP resistance in gastric cancer cells via activation of MEK/ERK and PI3K/Akt pathways.

### Reciprocol activation between PAK4 and PI3K/Akt pathway

**Figure 5:**
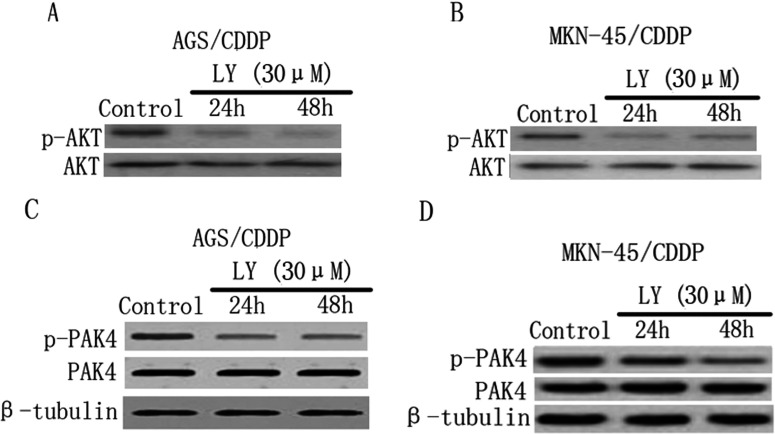
Inhibition of PI3K/Akt pathway reduces PAK4 activity in CDDP-resistant gastric cancer cells (**A** and **B**) The effects of the PI3K inhibitor LY294002 (LY) on both phosphorylated and total Akt expression levels were determined by Western blotting at the indicated time points in AGS/CDDP and MKN-45/CDDP cells, respectively. (**C** and **D**) The effects of the LY on both phosphorylated and total PAK4 expression levels at the indicated time points in AGS/CDDP and MKN-45/CDDP cells, respectively.

As confirmed above, inhibition of PAK4 activity led to suppression of both MEK/ERK and PI3K/Akt pathways. To dissect these pathways positively regulate PAK4 activity as well, we inhibited these pathways with their specific inhibitors. Figures 5(A) and 5(B) showed the potent inhibitory effect of PI3K inhibitor LY294002 (30 μM) on Akt activity at the indicated time points in AGS/CDDP and MKN-45/CDDP cells, respectively. In both AGS/CDDP (Figure 5C) and MKN-45/CDDP cells (Figure 5D), LY294002 (30 μM) treatment could inhibit PAK4 activity as the levels of phosphorylated PAK4 were reduced compared with control at the indicated time points. However, inhibition of PI3K/Akt pathway exerted no effects on PAK4 expression in these cells, as the total PAK4 levels were not changed after LY294002 treatment. Unlike the effects of LY294002, inhibition of MEK/ERK pathway with PD98509 (30 μM) had no influence on both PAK4 activity and expression in these cells (results not shown). Therefore the data together indicate that PAK4 and PI3K/Akt pathway reciprocally activate each other in CDDP-resistant gastric cancer cells.

### PAK4 confers CDDP resistance in gastric cancer cells *in vivo*

**Figure 6:**
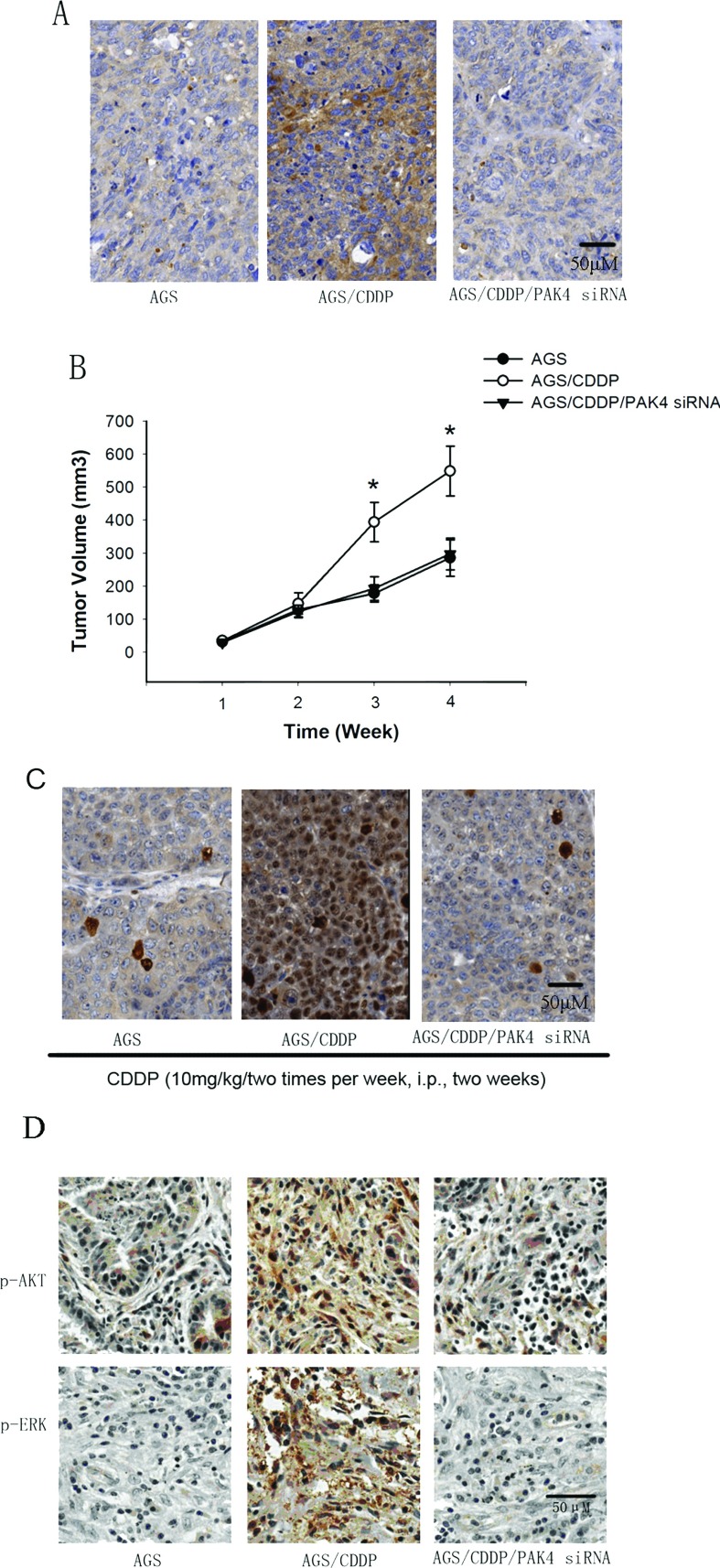
Effects of PAK4 siRNA on CDDP resistance in gastric cancer cells *in vivo* (**A**) Immunostaining of HtrA1 expression in AGS, AGS and AGS/CDDP/PAK4 siRNA xenograft tumours. (**B**) Tumour size was measured every two days for the indicated period. All mice were received CDDP treatment. *P* value was determined by log-rank test. Ten mice per group, mean±S.D. (**C**) The Ki67 staining per field from paraffin-embedded sections of AGS, AGS and AGS/CDDP/PAK4 siRNA tumours determined by immunohistochemistry. (**D**) p-AKT and p-ERK expressions in tumour tissues of different groups were analysed for immunohistochemistry. All scale bars represent 50 μm.

It was critical to determine whether PAK4 confers CDDP resistance in gastric cancer cells *in vivo*. AGS cells, AGS/CDDP cells and AGS/CDDP cells harbouring PAK4 siRNA (~1×10^7^ cells each group) were subcutaneously inoculated into the right flank of 6-week-old nude mice. When tumours reached 80–100 mm^3^, all the mice were received CDDP administration (10 mg/kg per two times per week, i.p.). Figure 6(A) showed that before CDDP administration, the levels of PAK4 were significantly higher in AGS/CDDP tumour tissues than those in AGS group. PAK4 siRNA treatment dramatically reduced the levels of PAK4 in AGS/CDDP tumour tissues. Figure 6(B) showed that the average tumour volume in the AGS/CDDP group was significantly larger than in the AGS group after 2-week CDDP treatment (*P*<0.05). However, no statistic differences were found in the tumour volume between AGS group and AGS/CDDP/PAK4 siRNA group. To further confirm the effect of PAK4 on CDDP efficacy, sections from tumours were subjected to Ki67 staining. The results showed that after 21-day CDDP treatment, AGS/CDDP tumours showed more Ki67 staining than AGS tumours, and PAK4 siRNA significantly reduced Ki67 staining in AGS/CDDP cells *in vivo* (Figure 6C). These results indicate that PAK4 knockdown can overcome CDDP resistance in gastric cancer cells *in vivo.* To explore the mechanism underlying PAK4-induced CDDP resistance *in vivo*, we performed immunohistochemical staining assay to determine the effect of PAK4 siRNA on the levels of p-AKT and p-ERK in AGS/CDDP tumour tissues. When tumours reached 80–100 mm^3^, the expression of p-AKT and p-ERK were determined. As shown in Figure 6(D), the levels of p-AKT and p-ERK were significantly increased in AGS/CDDP tumour tissues compared with the AGS group, and PAK4 siRNA treatment dramatically reduced the levels of p-AKT and p-ERK in AGS/CDDP tumour tissues.

## DISCUSSION

PAK4 has been confirmed to be correlated with the development and progression of various tumour cells. PAK4 kinase is required for oncogenic transformation of MDA-MB-231 breast cancer cells in nude mice *in vivo* [18]. Stable knockdown of Pak4 in ovarian cancer cell lines reduces cell migration, invasion and proliferation [19]. HGF (human growth factor)-driven prostate cancer cell migration involved activation of PAK4–LIMK1 pathway [20]. Recently, Park et al. [21] reported that PAK4 enhanced survival and decreased apoptosis following chemotherapy in prostate cancer. In gastric cancer, PAK4 overexpression was found in four (8.1%) of 49 metastatic cancer specimens and none of the four patients with PAK4(+) responded to capecitabine/cisplatin chemotherapy [15]. Such studies suggest a potential role of PAK4 in cancer chemosensitivity. In the present study, we first established two CDDP-resistant gastric cancer cell lines–AGS/CDDP and MKN-45/CDDP. In these cells, we found that PAK4 levels were significantly higher than those in their parental cells. Inhibition of PAK4 activity or knockdown of PAK4 expression reversed CDDP resistance. These results demonstrate that PAK4 induces CDDP resistance in gastric cancer cells, thus revealing a novel role of PAK4 in tumour biology. However, as PAK4 overexpression exists in multiple lines of cancer cells, whether such effect appears non-specific to various cancer cells needs further explored.

Constitutive activation of PI3K/Akt pathway [22] and MEK/ERK pathway [23] has been found in a variety of malignances. Inhibition of PI3K–Akt pathway increases CDDP efficacy in many cancer cell lines including gastric cancer cells [24–26]. Constitutive activation of PI3K–Akt pathway confers cancer cell resistance to many chemotherapy agents [27]. However, the role of MEK/ERK pathway in CDDP chemosensitivity in different cancer cell lines is inconsistent. Suppression of MEK/ERK pathway enhances CDDP resistance in human cervical carcinoma cells through activation of NF-κB (nuclear factor κB) pathway [28], whereas Sinnberg et al. [24] reported that inhibition of MEK/ERK pathway has no effect on CDDP cytotoxicity in melanoma cells. In the present study, we found that inhibition of PI3K/Akt or MEK/ERK pathway alone can both enhance CDDP efficacy in CDDP-resistant cells. Thus, combined with other findings, our data suggest that the roles of constitutive activation of PI3K/Akt and MEK/ERK pathways in chemosensitivity among various cancer cells are complicated. Pharmacological intervention of these pathways to enhance CDDP efficacy should be based on certain cancer cells.

PAK4 overexpression has been shown to promote ovarian cancer cell migration and invasion in a c-Src and MEK-1 kinase-dependent manner, and induce cell proliferation through the Pak4/c-Src/EGFR (epidermal growth factor receptor) pathway [19]. Lu et al. [29] reported that PAK4 regulates endometrial cancer cell migration and invasion, involving the ERK1/2 pathway-mediated MMP-2 (matrix metalloproteinase 2) secretion. In this study, we found that PAK4 induces CDDP resistance in gastric cancer cells, mainly through activation of PI3K/Akt and MEK/ERK pathways. These data together suggest that PAK4 exerts different cellular functions via different mechanisms. Furthermore, as mentioned above, constitutive activation of PI3K/Akt pathway and MEK/ERK pathway exists in a variety of malignances. However, the comprehensive mechanisms remain unclear. In this study, our results that inhibition of PAK4 leads to suppression of PI3K/Akt and MEK/ERK pathways suggest that PAK4 overexpression may contribute to such constitutive activation.

Interestingly, similar with other studies [30,31], we found that PI3K/Akt pathway positively regulates PAK4 activity, while it has no influence on its expression. These findings indicate that there exists a reciprocal positive regulation between PAK4 activity and PI3K/Akt signalling in CDDP-resistant cancer cell. This finding rules out the possibility that PI3K/Akt pathway regulates PAK4 activity through modulation of PAK4 expression. A possible explanation for such regulation may be consistent with that PAK1 can be activated through direct interaction with PI3K [32] and can be indirectly activated by Akt [32]. The reciprocal activation between PAK4 and PI3K/AKT pathways may represent a positive regulatory circuit that mutually reinforces the PAK4 and Akt activities to further augment the CDDP resistance in gastric cancer.

It will be important to determine the effect of PAK4 on CDDP resistance in gastric cancer cells *in vivo*. Furthermore, it remains to be investigated whether the role of PAK4 in CDDP resistance development is non-specific to certain tumour cells. Notwithstanding these limitations, our study does indicate that PAK4 confers CDDP resistance via activation of MEK/ERK and PI3K/Akt pathways and there exists a reciprocal activation between PAK4 and PI3K/AKT pathways in gastric cancer cells *in vitro*. Therefore PAK4 may be a potential target for adjuvant chemotherapy in gastric cancer.
